# Rice Straw Tissue Preparation for Reproducible Electron Microscopy Imaging and Analysis

**DOI:** 10.1002/cpz1.70262

**Published:** 2025-12-16

**Authors:** Mahta Mohamadiaza, Naser Farrokhi, Pär K. Ingvarsson, Asadollah Ahmadikhah, Mehdi Jahanfar

**Affiliations:** ^1^ Department of Cell & Molecular Biology, Faculty of Life Sciences and Biotechnology Shahid Beheshti University Tehran Iran; ^2^ Department of Plant Biology Swedish University of Agricultural Sciences Uppsala Sweden

**Keywords:** anatomical traits, dry sample preparation, image processing, image quantification, *Oryza sativa*, plant cell wall

## Abstract

Common problems in biological sample processing for scanning electron microscopy (SEM) include cell collapse and destruction. To overcome the challenges surrounding SEM micrograph preparation, dried rice stems were used to develop a specific set of protocols for processing dried plant samples. Dried stems are rehydrated with a glycerol solution and fixed in formalin‐acetic‐alcohol to avoid cell wall collapse or organ distortion. The protocols detailed here comprise the first published method for preparing SEM images of dried plant tissue. The protocols offer a cost‐effective approach to obtaining high‐quality micrographs, facilitating the reconstruction of growth processes and the study of plant cell wall features. © 2025 The Author(s). Current Protocols published by Wiley Periodicals LLC.

**Basic Protocol 1**: Pretreatment and preparation of rice straw samples at the heading stage

**Basic Protocol 2**: Paraffin infiltration and embedding

**Basic Protocol 3**: Preparation of microscopic sections

**Basic Protocol 4**: Transferring, adhering, and expanding sections on slides

**Support Protocol**: Preparation of gelatin slides before sectioning to affix samples

**Basic Protocol 5**: Preparation of samples for SEM imaging

**Basic Protocol 6**: SEM analysis

**Basic Protocol 7**: Processing and analysis of SEM images using ImageJ software

## INTRODUCTION

Scanning electron microscopy (SEM) is an ideal technique for examining plant surfaces at high resolution (Pathan et al., 2010). In biology, the use of SEM has been extended to studies of structural evolution, comparative morphology, organ development, and characterization of populations or species (Endress et al., 2000). SEM uses electrons ejected from the surface of the specimen to reproduce the topography in a high‐vacuum environment (Everhart & Thornley, 1960).

Studies involving SEM have focused on inferring structural characteristics and reconstructing growth processes (Bello et al., 2017; Endress, 1972; Tucker, 1975). For example, new structural characteristics relevant to the taxonomy and systematics of a wide range of organisms have been discovered from SEM images (Fannes et al., 2015). Furthermore, the reconstruction of growth processes using SEM has made it possible to study growth defects (Benhamou et al., 1999), plant root physiology (Ratnayake et al., 2012), and comparative development of wild and mutant individuals (Singh et al., 2014) as well as to conduct structural studies of organ development (García et al., 2015) or entire life cycles (Xiang et al., 2015). Thus, SEM has merits in both static analysis (capturing a snapshot of the structure to determine the geometric and chemical properties of the sample at a specific time) and dynamic analysis (scientific videography to understand how a sample changes over time or under specific conditions) of biological samples (Table 1).

**Table 1:** Brief Overview of the Differences between Static and Dynamic Analyses

Property	Static	Dynamic
Time	Single image or still moment	Timeline or video
Sample state	Stable and unchanging	Changing (thermal, mechanical, chemical)
Main objective	Measurement and analysis of the instantaneous structure	Monitoring process and behavior over time
Additional equipment	No need	Usually requires *in situ* systems (stage and special enclosure)

Environmental SEM (ESEM) (Mendoza et al., 1993) has been used for wet samples. However, delicate samples may still be compromised, especially under the low‐vacuum conditions required by the instrument. Thus, to avoid loss of valuable morphological information, the samples need to be prepared meticulously (Bello et al., 2017).

**Figure 1:**
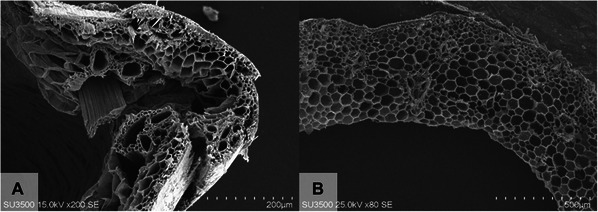
Comparison of recorded images of the cell wall structure of dry stem (straw) before (**A**) and after (**B**) applying the sample preparation protocol to rice straw. Scale bars: (A) 200 µm and (B) 500 µm. Photos taken with an SU3500 SEM system.

Plant tissues exhibit a diverse range of types, shapes, structures, and compositions. Moreover, lab‐to‐lab variations are inevitable due to the diversity in individual skills and types of equipment. Therefore, the constant development of new/modified techniques in plant sample preparation is necessary for better visualization under electron microscopes. To date, various methods have been proposed for preparing samples for SEM imaging, most of which have been based on studying wet samples or have recommended the use of wet samples to obtain the desired image with good resolution. To the best of our knowledge, no specific protocol has been proposed for preparing old, dried plant samples. This has led to the exclusion of these samples from many studies. This article aims to provide an efficient and repeatable method for accessing the information hidden in such samples and maximizing their potential. By returning these samples to the research workflow, valuable information will be made available to researchers. For the first time, this article specifically describes a specialized method for preparing dried stem samples, regardless of age, for SEM (Fig. 1).

This set of protocols can be used to prepare samples such as herbarium specimens, samples that cannot be studied wet for any reason (such as due to a large sample volume, which may cause mold to form by the time of imaging, or due to moisture destroying the sample or part of it). These protocols can also be used in cases such as having missed the optimal time for imaging the sample (in cases where the researcher is unable to prepare the sample at a specific time and postpones preparation to another time) and needing to prepare documentation of the samples for future studies. To validate and verify the reproducibility of the proposed protocols, dried stems (straw) from 147 rice samples collected from different geographical locations worldwide were used to prepare SEM micrographs. Phenotypic data obtained by quantifying cell wall structural information from SEM images of the studied stem samples can be used in studies such as genome‐wide association studies (GWASs) (unpub. observ.). Our data were compared with data from previously reported methodologies, and the results were illustrative of our technique's greater efficiency in preserving cellular shape and structure. Our method for obtaining accurate and high‐quality images (in terms of no structural breakage and preserving the intact structure) provides a step‐by‐step approach for quantifying image information, and this quantitative information can be utilized in applications such as machine learning, neural networks, and deep learning, given sufficient data. Here, the methods of pretreatment and preparation of rice straw samples collected at the clustering stage are described step by step, in three parts: (1) rehydration, (2) fixation and washing, and (3) dehydration and clearing of samples (Basic Protocol 1). Paraffinization and preparation of paraffin blocks from the samples are described in Basic Protocol 2; preparation of microscopic sections, in Basic Protocol 3; preparation of gelatin slides, in the Support Protocol; and passage and fixation of the samples, in Basic Protocol 4. Basic Protocol 5 describes the step‐by‐step preparation of samples for SEM imaging, and Basic Protocols 6 and 7 represent, respectively, how to acquire SEM images and how to process and analyze the SEM images using ImageJ software.

*CAUTION*: Use properly labeled glass containers throughout the experiment. Many of the solutions used in this article are corrosive (toluene) and/or toxic (various types of alcohols).

## Basic Protocol 1 PRETREATMENT AND PREPARATION OF RICE STRAW SAMPLES AT THE HEADING STAGE

Monocot tissues, such as those found in rice, are brittle due to the high amounts of silica, which can damage the cells during sectioning. This protocol softens the stem tissue as much as possible, facilitating the sectioning process (Basic Protocol 3). Given that the samples are thoroughly dried, this protocol is crucial. Previously, liquid replacement with low‐vapor‐pressure liquids, such as glycerol, has been proposed as a simple method for preparing biological samples for imaging by conventional SEM due to increased conductivity and water miscibility (Ensikat & Barthlott, 1993; Pathan et al., 2010). Inspired by the use of glycerol, we describe the first step in the preparation of dried rice stem tissue.

### Materials

Collected dried plant materials [with stems ∼2 cm long; dried for a long time (≥1 year); e.g., rice straw samples separated from third internode, International Rice Research Institute (IRRI), Philippines]
50% (v/v) glycerol solution
Distilled water (dH_2_O)
Fixation solution (formalin‐acetic‐alcohol, or FAA; see recipe; make fresh)
50% and 70% (v/v) ethanol

Labeled 15‐ml glass vials (see Video 1)
Tweezers
Paper towel

**Video 1:** Demonstration of how to label the glass vials used in all stages of implementation. This is particularly important to follow.

#### Sample rehydration

1 Divide the collected dried plant materials [with stems ∼2 cm long; dried for a long time (≥1 year)] into small pieces of a few centimeters each and put them in labeled 15‐ml glass vials containing a 50% glycerol solution for 20 days at 4°C. Ensure that the solution covers the tissue sample.
*IMPORTANT NOTE*: Regularly inspect the samples during this period. If you see the traces of color changes in the solution containing the samples, transfer them to another container with the same contents.

#### Sample fixation and washing

2 After 20 days of rehydration, wash the samples first with running water and then with dH_2_O. Immerse in fixation solution and incubate for 5 days at 4°C.
  See Video 2. The primary purpose of fixing the samples is to alter the physical and chemical properties of the cells in a manner that preserves the cell structure and intracellular characteristics during the sectioning process. A more wooden texture requires more extended fixation periods (with a maximum of twice the time mentioned above, i.e., 10 days). The stiffness of tissues is considered positive up to a certain point, as it facilitates the preparation of thin sections. However, if this stiffness (referring to the amount of stiffness and shrinkage of the tissue that occur after the fixation solution is absorbed) exceeds a certain level, it causes disintegration and tissue failure. Therefore, it is essential to adhere strictly to the specified time for this stage. Consequently, it is recommended to use the 5 days mentioned above for rice samples. If the stem tissue you are studying is thicker and belongs to a plant other than rice, it is recommended to double this period.

**Video 2:** Overview of how to wash samples and how to transfer samples from one solution to another, which is related to Basic Protocol 1.

#### Washing, dehydration, and clearing of samples

3 Remove samples from the fixation solution using tweezers.
4 Wash samples under running water.
5 Wash with dH_2_O for 2 to 3 min.
6 Gently place samples on a paper towel to remove excess water.
7 Place the samples in 50% ethanol for 10 min.
8 Transfer the samples to 70% ethanol and incubate for 5 days at 4°C.
  Fixed material can be stored indefinitely in 70% ethanol.

## Basic Protocol 2 PARAFFIN INFILTRATION AND EMBEDDING

Paraffin infiltration enables the preparation of very thin sections using a microtome (Basic Protocol 3). For this reason, plant tissues (here, the stem) are impregnated with a strengthening material such as paraffin after the fixation stage (Basic Protocol 2) to obtain sufficient strength for sectioning. Due to the cellulosic wall of plant tissues, the penetration of paraffin into the tissue is relatively slow, so it takes a few days for the paraffin to penetrate. The longer this time is, the better the sections that will be obtained. The time required for the paraffin to penetrate the tissues completely varies depending on the type of tissue sample. The samples must be kept in the third paraffin bath in the oven until they are completely clarified. The oven temperature must be controlled during the incubation period to prevent it from dropping below 70°C, which would prevent the paraffin from solidifying and thus prevent it from penetrating the tissues. At this stage, paraffin penetration into the tissue results in whiter samples, indicating that the paraffin has successfully infiltrated the tissue.

### Materials

Paraffin
90% (v/v) and 100% ethanol
Toluene
Samples from Basic Protocol 1

Oven
Labeled 15‐ml glass vials (see Video 1)
Tweezers
Chemical hood
L‐shaped brass mold pieces
Fine tweezers, preheated over flame
Metal loop, heated over alcohol lamp

1 To melt the paraffin, first set the oven temperature to 70°C (the melting point of the paraffin granules). Then, pour the paraffin into a labeled 15‐ml glass vial and place it in the oven until the paraffin has melted.
2 Proceed with ethanol and toluene 1 treatment of the samples from Basic Protocol 1 according to Table 2 (see Videos 3 and 4).
  To dehydrate samples, alcohol solutions of increasing concentrations (50%, 70%, 90%, and 100%) can be used. Transferring samples from one alcohol solution to another does not require a water wash step.
*CAUTION*: Because toluene is a strong solvent, the toluene incubations should be performed with extra care and precision. When working with toluene, refer to the safety sheet and work under a fume hood. Use glassware due to the corrosive nature of toluene.

**Table 2:** Steps and Time Required for Dehydrating and Clarifying Plant Samples (Stems) and Preparing for Embedding

Date	Steps	Time
The sixth day	90% ethanol	1 hr at 25°C
	100% ethanol 1	1 hr at 25°C
	100% ethanol 2*^a^*	1 hr at 25°C
The seventh day	Toluene 1	1 hr at 25°C
	Toluene 2*^b^*	2.5 hr at 25°C
	First paraffin bath	2 hr at 70°C
	Second paraffin bath	14 hr at 70°C
	Third paraffin bath	7 days at 70°C

^*a*^ To experiment on the next day, the samples are placed at 4°C. The samples are removed from the 100% ethanol with tweezers and placed in toluene 1 on the following day.

^*b*^ This step makes the tissue more transparent, and the clearer the tissue, the better the cuts that can be made.

**Video 3:** Transfer of samples between alcohol solutions. This video points out that Basic Protocol 2 does not require a wash step when transferring samples between alcohol solutions.

**Video 4:** Review of how to perform the steps listed in Table 2. The steps for transferring the sample to toluene should be performed under a chemical hood, with caution. Any plastic will melt due to toluene's corrosive properties, even the experimenter's gloves.

**Figure 2:**
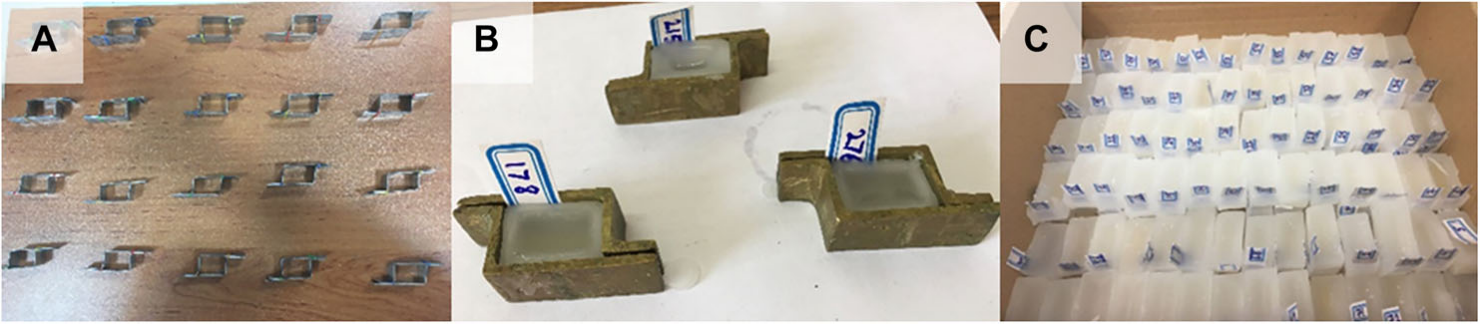
Molds and paraffin blocks. (**A**) Paraffin molds made of brass; each assembled mold contains two pieces of surface‐smoothened, bent metal in the shape of the Latin letter L. (**B**) Labeled paraffin molds containing the desired sample. (**C**) Paraffin blocks containing different stem specimens that have been removed from the molds.

3 Immerse the samples in toluene 2 solution for 2.5 hr (see Video 4).
4 Transfer to the 70°C oven so that no breakage in paraffin takes place.
5 Remove the samples from the toluene 2 with tweezers under a chemical hood.
6 Place the samples in the first paraffin bath.
7 Immediately transfer to a 70°C oven for 2 hr.
8 Transfer the samples to the second paraffin bath at 70°C for 14 hr.
9 Transfer the samples to the third paraffin bath at 70°C for 7 days.
10 After the samples are entirely covered with paraffin, place two L‐shaped brass mold pieces on a flat surface as shown in Figure 2A.
  Molds can be made of metal (preferred), strong plastic, wood, or even glass. The edges and sides of the molds must be completely smooth; this way, the molds can be stably placed on a flat surface so that the molten paraffin does not leak.

11 Pour some melted paraffin into the assembled mold and wait until it is half‐set.
  To keep the paraffin molten during the embedding process, place the paraffin jars on a heater.
12 Place the first sample in the mold with fine tweezers preheated over a flame, keeping the proper orientation (see Video 5).
  A high operating speed is necessary to ensure that samples are entirely flat and laid in the paraffin in the proper orientation so that axial cuts can be made later at the sectioning stage.

**Video 5:** Depiction of the sample casting steps in Basic Protocol 2. The key point here is to embed the label inside the paraffin mold to prevent the sample from being misidentified after it is placed in the paraffin block. To prepare the most desirable paraffin block from the desired sample, the speed of the operation and the temperature of the paraffin when pouring into the mold are of particular importance, as well as ensuring the absence of bubbles during this process. If bubbles are produced, remove them with a metal loop heated over an alcohol lamp.

13 Fill the mold with molten paraffin immediately (Fig. 2B). Remove any formed air bubbles with a metal loop heated over an alcohol lamp.
  Filling in intervals leads to a layered paraffin block that will be crushed during block sectioning in Basic Protocol 3.
14 Let the block cool down, until the paraffin hardens, and remove the mold slowly (Fig. 2C).
  The sample specifications and the date of preparation can be written on a label and placed inside the paraffin at the edge of the mold, right before the paraffin block is completely cooled (see Video 5).

## Basic Protocol 3 PREPARATION OF MICROSCOPIC SECTIONS

The thickness of the sections taken from the tissue varies depending on the purpose of the study and the imaging type. For example, the most suitable thickness for studying tissues under an optical microscope is 7 µm. However, according to our experience, the thickness of the tissue does not have a great impact on the intactness for imaging with SEM; only the vertical sectioning plays a decisive role in not having any visible angle. To prepare smooth sections from samples fixed in a paraffin block (Basic Protocol 2), it is necessary to perform a trimming operation so that the sample can be placed on the sectioning surface.

### Materials

Paraffin‐embedded samples (see Basic Protocol 2)

**Figure 3:**
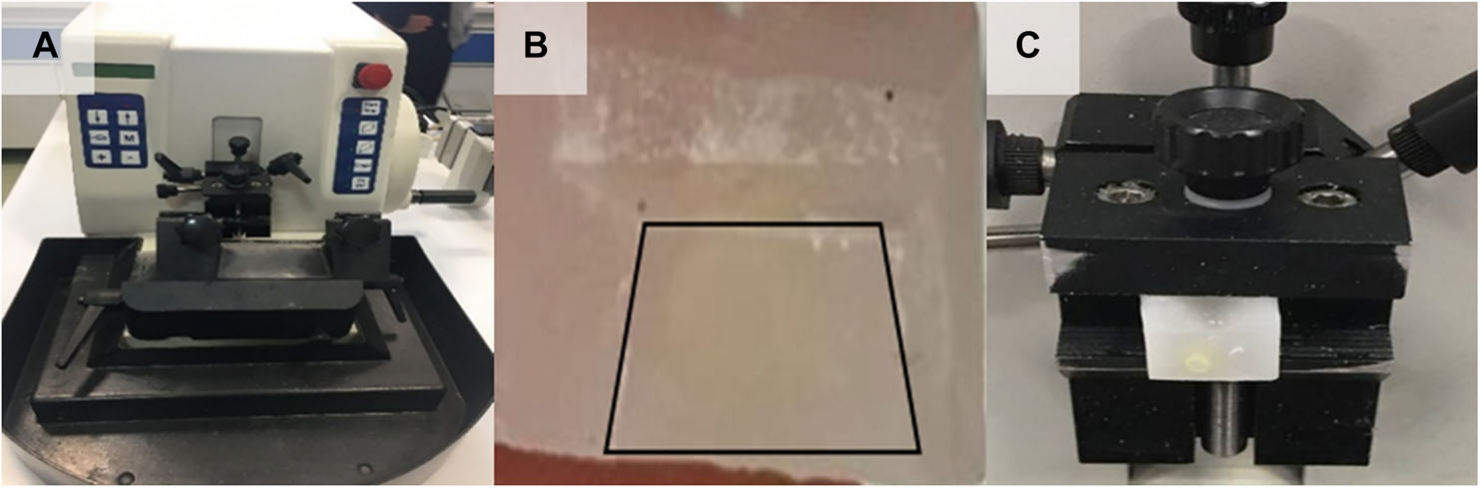
Microtome and how to place the sample in it. (**A**) SLEE CUT 6062 rotary microtome. (**B**) Paraffin block trimmed in the shape of a trapezoid. (**C**) Paraffin block fixed on a microtome.

Scalpel
Rotary microtome (SLEE, CUT 6062; Fig. 3A)
Additional reagents and equipment for transferring sections to slides (see Basic Protocol 4)

**Figure 4:**
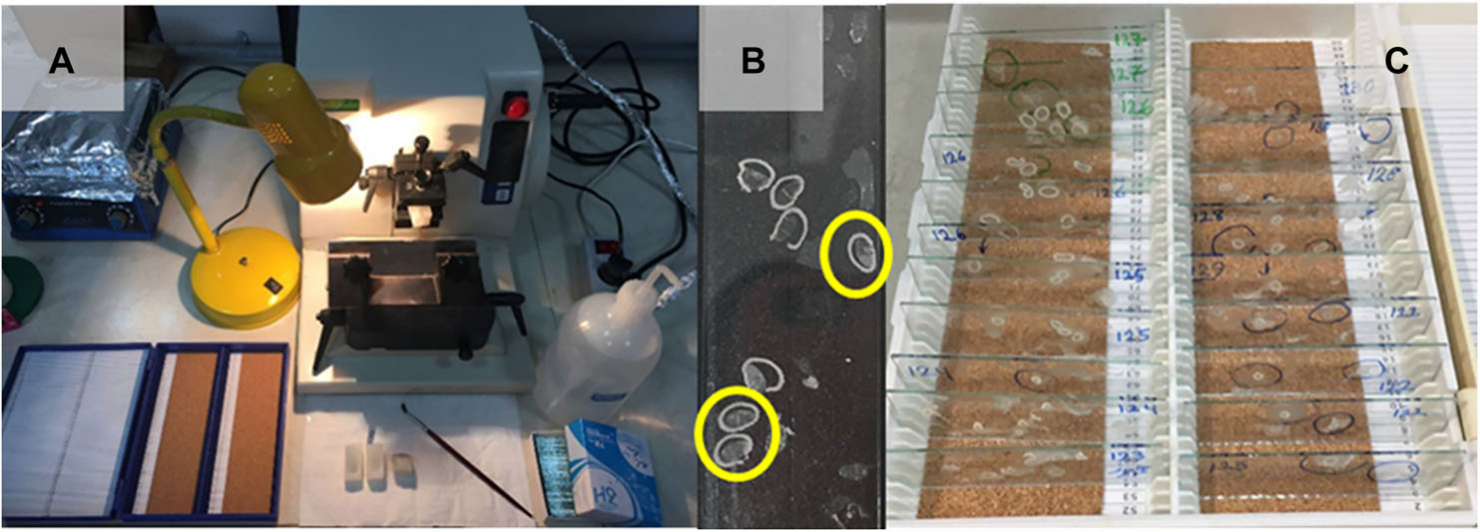
Slide preparation. (**A**) The items needed include a slide, brush, specimen fixed in a paraffin block, reading lamp, heater, and microtome and distilled water. (**B**) Slide containing a cross‐section of a rice stem sample. (**C**) Slides prepared from labeled samples should be placed at room temperature for 1 week to dry.

1 Use a scalpel to remove the excess paraffin around the paraffin‐embedded sample, forming a trapezoid (Fig. 3B).
2 To prepare smooth sections, first smooth the surface of the sample manually with the scalpel.
3 Place and fix the paraffin block on the rotary microtome and ensure that the angle of the microtome blade relative to the sample is adjusted to create vertical sections. Use screws to make the block parallel to the blade right in the middle (Fig. 3C).
  Before working with the microtome, it is better to heat its blade a little, for example, using a reading lamp (with a standard bulb that generates heat; Fig. 4A). In particular, the head of the lamp is bent toward the blade to warm the blade with the gentle heat generated. This allows for ease of cutting when the sample hits the cutting blade.
  The angle of the blade relative to the paraffin block containing the sample must be chosen correctly.

4 Section the sample, with the big face of the trapezoid facing down (Fig. 3B). Before creating the main sections, prepare a few preliminary sections to ensure that the sample's surface is smooth. Generate the main sections from the sample with only one movement by rotating the microtome handle to create fine sections with a thickness of 30 µm, transferring the sections to slides according to Basic Protocol 4.
  Make the serial cuts so that the cut surface is completely smooth, with no excess paraffin (Fig. 3C).
  To obtain perfect strips of cross‐sections, the blade must be completely clean, sharp, dry, and toothless. The razor blade can be cleaned using a cotton ball dipped in toluene or ethanol, but it is preferable to use a disposable blade.

## Basic Protocol 4 TRANSFERRING, ADHERING, AND EXPANDING SECTIONS ON SLIDES

This protocol describes how to adhere and expand the sample (Basic Protocol 3) onto the slides (Support Protocol), which is done with the help of gentle heat from a slide warmer. Given that multiple sections of a sample can be placed on a labeled slide, the best sample for SEM imaging can be selected and marked while observing the sample under an upright microscope. See Video 6 for a demonstration of microtome sectioning and mounting and spreading of samples on slides.

**Video 6:** Microtome sectioning and mounting and spreading of a sample on a slide, as well as taking SEM images.

### Materials

dH_2_O
Sample sections (see Basic Protocol 3)

Dropper
Gelatin slides (see Support Protocol) or uncoated slides
Wet brush
Slide warmer
Paper towel
Upright microscope (Zeiss Primostar)

*NOTE*: In our experience, sections prepared from rice straw do not need slide gelatinization to stick to the slide, so uncoated slides can be used.

**Figure 5:**
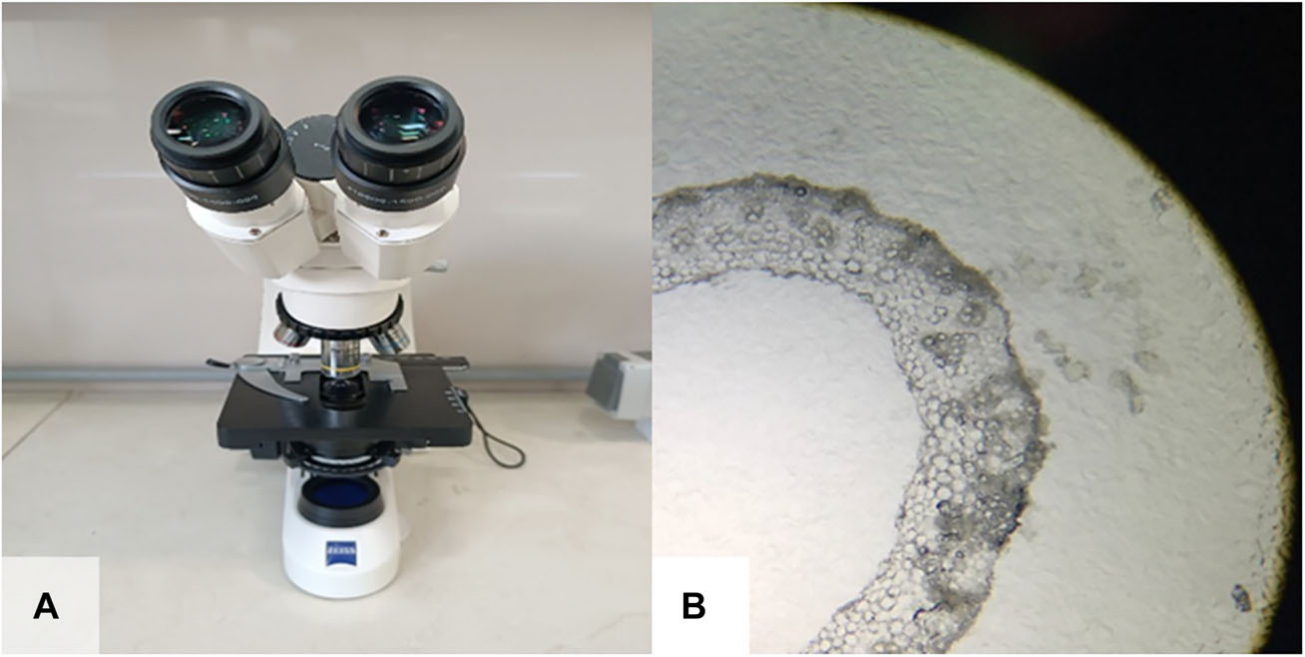
Microscopy of sectioned samples. (**A**) Upright microscope. (**B**) Image of a desirable cross‐section (rice straw).

1 Use a dropper to place a few drops of dH_2_O on the gelatin or uncoated slide after choosing the desired section.
2 Transfer the sample section from the microtome blade to the slide with a wet brush.
3 Adhere and expand the section on the slide. Use gentle heat from the slide warmer, moving the slide back and forth over the warmer, to gradually open the folds of the sample. Remove excess water on the slide using a paper towel.
  Several sections from the same sample can be placed on a slide to select the best slice under the upright microscope in step 5 (Fig. 4B).
*CAUTION*: Be careful to set the heater to a temperature that is warm enough to be comfortably touched. Higher temperatures will also cause the water to quickly condense on the sample, potentially causing it to burn.
4 For complete sample‐slide attachment, keep the slide at room temperature for 1 week (Fig. 4C).
5 To ensure that the desired cut is obtained, observe the slide containing sections from the sample using an upright microscope (Fig. 5A and 5B; see Video 6).
  The prepared slides can be stored for extended periods in a dry environment (Fig. 4C).

## PREPARATION OF GELATIN SLIDES BEFORE SECTIONING TO AFFIX SAMPLES

While preparing cross‐sections of the sample (Basic Protocol 3), the cut sample is placed on a slide for fixation (Basic Protocol 4). This protocol describes how to prepare gelatin slides for this purpose (see Video 7). However, our experience with the process shows that paraffin, which melts when the sample is heated, can also be used as an adhesive to fix the sample to the slide (Fig. 4B and 4C; see Videos 6 and 7).

**Video 7:** Demonstration of the steps of the Support Protocol, which includes instructions on preparing gelatin slides.

### Materials

Gelatin solution (see recipe; make fresh)

Filter paper
Glass container
Slides
40°C heater (e.g., slide warmer)

1 Pour the gelatin solution through a piece of filter paper.
2 Pour the filtered solution into a glass container and immerse the slides in the solution for 20 min (see Video 7).
3 Remove the slides from the solution and place them vertically to remove excess solution and allow them to dry at 40°C for 48 hr.

## Basic Protocol 5 PREPARATION OF SAMPLES FOR SEM IMAGING

For SEM imaging, the samples on the slides (Basic Protocol 4) must be dewaxed, which means that the paraffin inside the tissues must be removed. Toluene solvent is used for this purpose.

### Materials

Samples on slides (see Basic Protocol 4)
Toluene

Chemical hood
Glass dropper
Paper towel
Upright microscope
Stereomicroscope

1 Wash the samples on slides in toluene for 40 min under a chemical hood. In particular, use a glass dropper to add toluene to the slide, followed by moving/tilting slide by hand to spread the toluene.
2 Remove excess toluene by allowing the slide to rest on one of its corners on a paper towel.
3 Repeat this process (steps 1 and 2) until the paraffin is completely removed from the tissue and the inside of the empty cells can be visually observed under an upright microscope.
*CAUTION*: Because toluene is a strong solvent that may cause the label to be washed off the slide, this step should be performed with extra care and precision. When working with toluene, refer to the safety sheet and work under a fume hood. Use glassware due to the corrosive nature of toluene.
*CAUTION*: To expedite and facilitate work at this stage, you can also use the gentle heat of a slide warmer (so gentle that when touching the surface, it does not feel hot to the touch). This method should not be attempted unless you are a professional.
4 Re‐check the sample using a stereomicroscope to ensure that the paraffin has been completely removed from the sample, leaving only the cell wall visible.

## Basic Protocol 6 SEM IMAGING

After successfully executing Basic Protocols 1 to 5, the sample is now ready for SEM imaging. This protocol describes how to prepare the sample for placement in the scanning electron microscope and finally image it.

### Materials

Deparaffinized samples (see Basic Protocol 5)
Gold

Tweezers
SEM pin stubs
Multiple pin stub holder topped with double‐sided carbon conductive paste
Desktop magnetron sputtering device
SEM system (SU3500, Hitachi)

**Figure 6:**
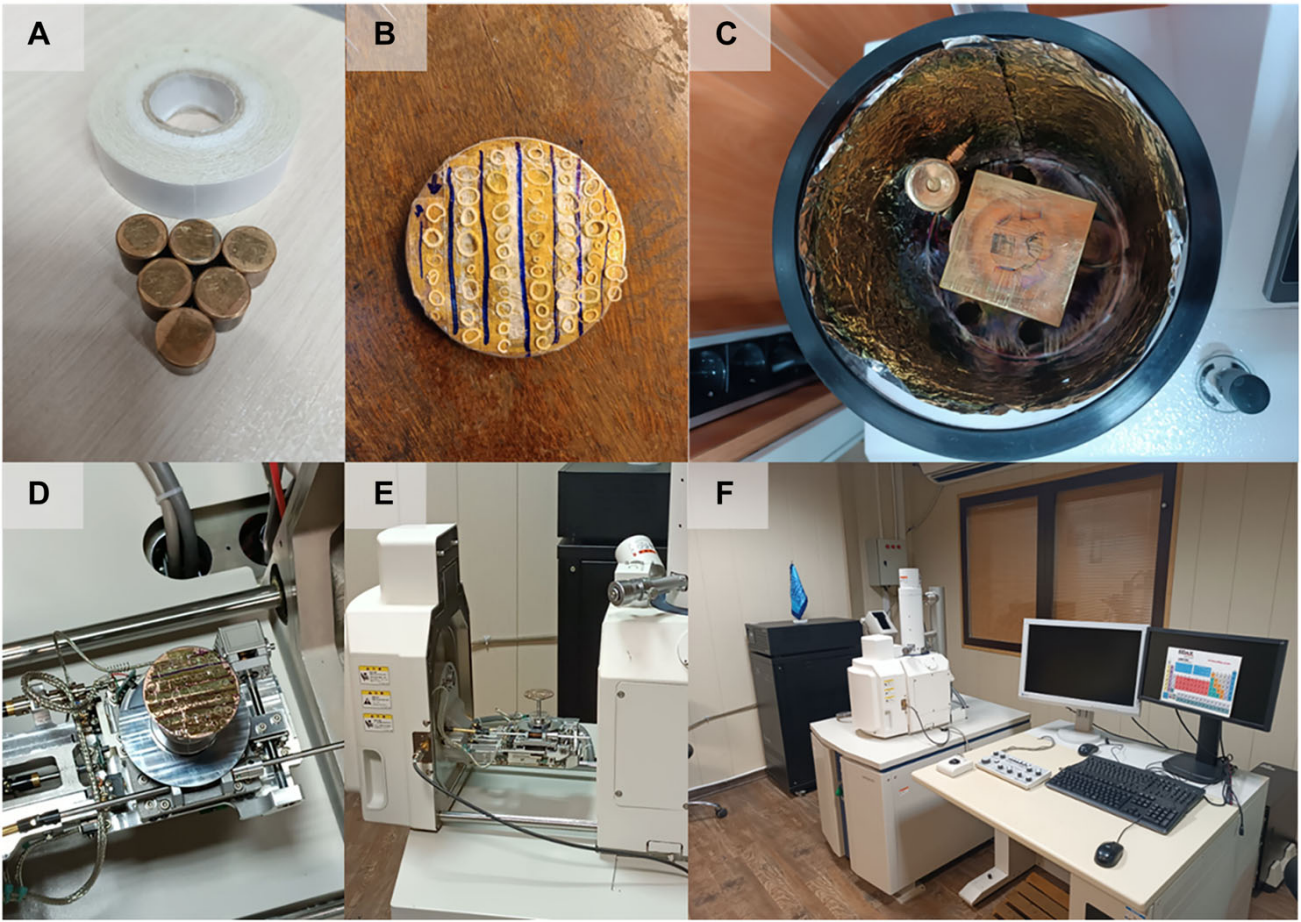
SEM imaging. (**A**) Multiple pin stub holder and carbon conductive paste. (**B**) Samples arranged on the holder based on the map to determine the reading direction for imaging. (**C**) Top view of the vacuum chamber. (**D**) Gold‐sputtered samples. (**E**) Placement of gold‐sputtered samples in the scanning electron microscope. (**F**) SEM imaging.

1 Using tweezers, slowly transfer the deparaffinized samples from slides to standard SEM pin stubs in a multiple pin stub holder topped with double‐sided carbon conductive paste (Fig. 6A).

2 Arrange the samples vertically and consecutively in the holder (Fig. 6B).
  The reading direction is marked with an arrow for tracking inside the scanning electron microscope.
3 Draw the map of the sample arrangement on paper.
4 Sputter gold on the samples using a desktop magnetron sputtering device (Fig. 6C and 6D).
5 Place the holder inside the SEM system (Fig. 6E) and image at the desired voltage (accelerating voltage = 25000 V) and magnification (Fig. 6F).

## Basic Protocol 7 PROCESSING AND ANALYSIS OF SEM IMAGES USING ImageJ SOFTWARE

**Figure 7:**
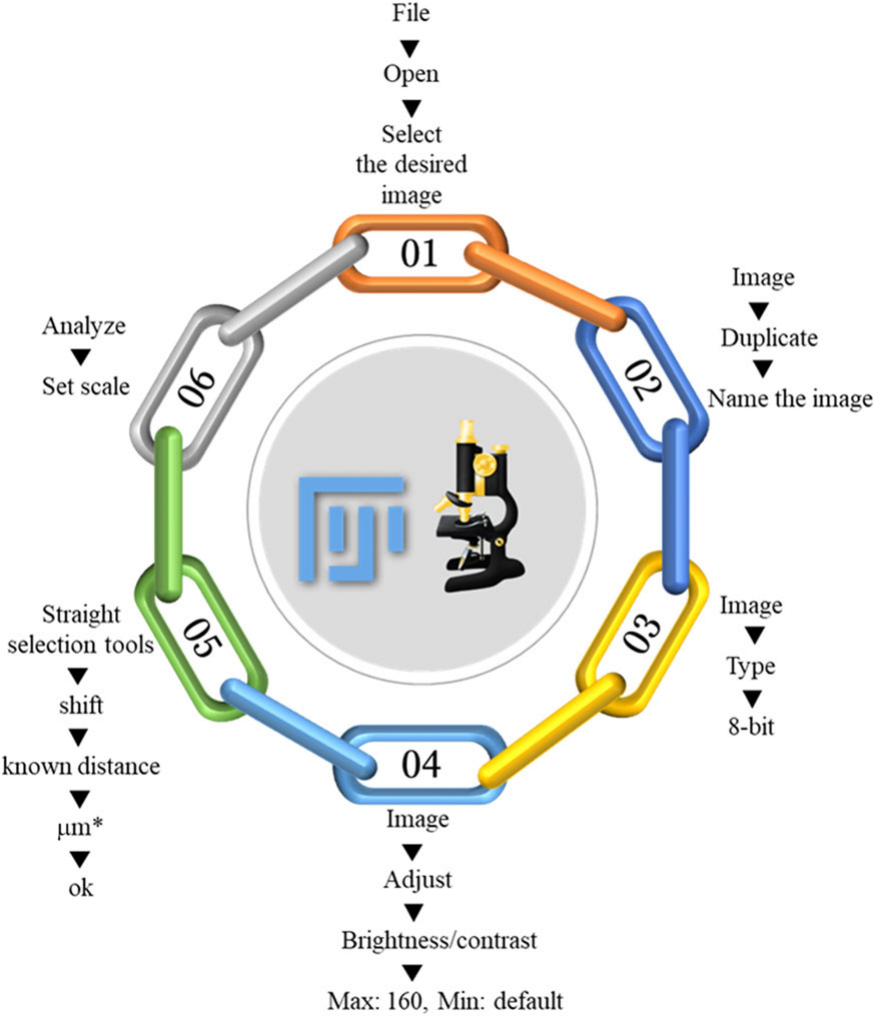
Schematic of image processing steps for quantification.

**Figure 8:**
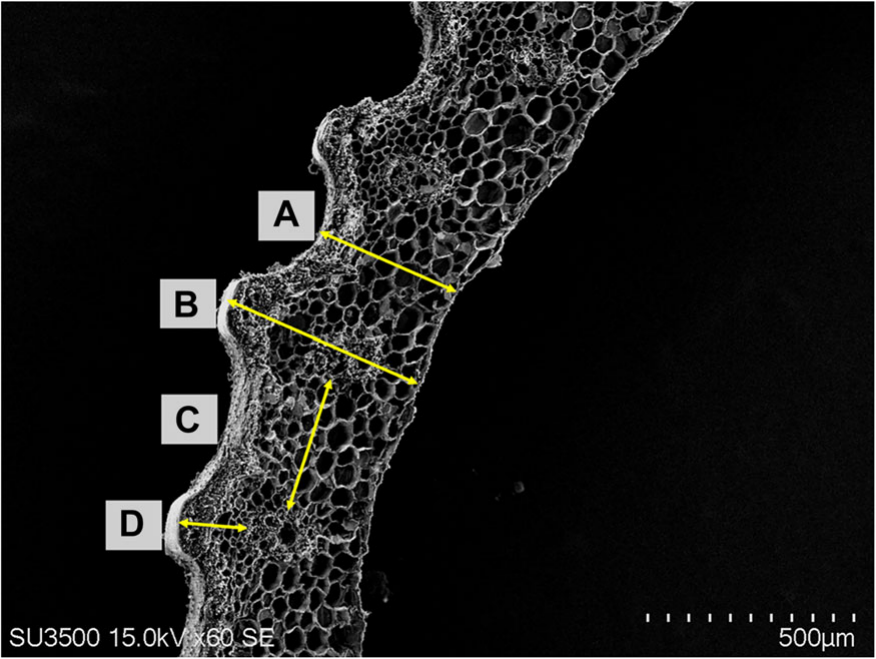
Measurement of different parts of the rice straw cell wall using ImageJ software. (**A**) The total thickness of the cell wall in the indented part. (**B**) The total thickness of the cell wall in the protruding part. (**C**) The distance between the vessels. (**D**) The distance between the vascular bundles and the end of the parenchyma (epidermis). Scale bar: 500 µm.

Until now, in most cases, the preparation of SEM images has been limited to visual observation and comparison of two samples: control and treated or wild and mutant. However, the most important question that has not been clearly answered so far is how to process the images and extract quantitative information for statistical comparisons. In this protocol, for the first time, an attempt has been made to accurately answer this question by explaining, step by step, the processing and quantification of images obtained from SEM. This method measures different parts of the image according to their scale. The data obtained can be used for further analyses (Fig. 7 and 8).

### Materials

SEM images from Basic Protocol 6
Fiji software (ImageJ, version 1.52; www.imagej.net/ij; Schindelin et al., 2012)
SPSS software (version 16.0)

1 To process the SEM images from Basic Protocol 6 and record the quantitative value of each trait using Fiji software (ImageJ, version 1.52), follow the steps shown in Figure 7.
2 Select “Set Measurement” from the Analyze menu. Mark the parameters to measure.
3 To record the measurement and display the recorded results page, select the “Measure” option from the Analyze menu.
4 Draw a straight line of the desired length on the image by pressing the Select and Shift buttons simultaneously (Fig. 8).
5 To save the results page information, select “File” and then “Save As” and save to CSV format.
6 Process quantitative features using SPSS software (version 16.0) to obtain the mean values of the traits (Jehanzeb et al., 2020; Zaman et al., 2022).

## REAGENTS AND SOLUTIONS

### Fixation solution (formalin‐acetic‐alcohol, or FAA)

To prepare 100 ml of this fixative solution, 90 ml of 50% (v/v) ethanol is mixed with 5 ml glacial acetic acid and 5 ml of 37% (w/v) formaldehyde under a chemical hood (Ruzin, 1999). Prepare fresh immediately before use if possible. Otherwise, store ≤1‐3 months at 4°C as long as there are no visible signs of precipitation or loss of clarity.

### Gelatin solution

Dissolve 5 g gelatin powder or sheet in 1000 ml dH_2_O with a glass stirrer at 37°C (to maintain the water temperature, you can put the water container on a hot plate). Add 0.5 g chromium potassium dodecahydrate (chrome alum) to dH_2_O and let it sit until the solution is completely homogenized, with no solid materials remaining (see Video 7). Filter the solution before use. Prepare fresh immediately before use if possible. Otherwise, store ≤1‐3 months at 2‐8°C as long as there are no visible signs of precipitation or loss of clarity. It is recommended to filter the solution again immediately before use, after bringing it to room temperature.

## COMMENTARY

### Background Information

To date, optimal protocols have been proposed for the preparation of young and delicate plant tissues, such as young flower meristems, for SEM imaging (Bello et al., 2017). Specific sample preparation is used for the visualization of leaves with specific types of electron microscopes (Pathan et al., 2010), as well as for the investigation of the composition of diverse cell wall polymers and the determination of acetate content in different rice varieties (Zhang et al., 2019). In most cases, cell disintegration and destruction, cell distortion, and inadequate preservation of the original tissue shape and structure, as well as the treatment of samples from humid microenvironments, have been cited as the most important challenges in preparing biological samples for observation with conventional SEM. Findings suggest that there is no universal procedure for processing plant tissue for SEM study (Pathan et al., 2010). This article presents a method for preparing dried rice straw samples by pretreatment with glycerol, along with sample fixation methods, and demonstrates the methods’ implementation with 147 different samples and validation by SEM. A method for image processing and quantification is also described to facilitate downstream analysis.

### Critical Parameters

Because the sample under study is dry, one of the most critical steps in the preparation process is sample placement in the 50% glycerol solution (Basic Protocol 1). It is recommended to check the sample regularly while it is in this solution. If you notice any color change in the solution, transfer the sample to a new container containing the same solution. Because half of the composition is water, there is a possibility of mold, even if it is stored in the refrigerator.

To achieve the desired cross‐sections of the desired sample, monitoring the penetration of paraffin into the tissue during paraffin embedding (Basic Protocol 2) is of utmost importance. Researchers are advised to ensure that the tissue does not become bleached due to paraffin absorption, which is easily detectable, before proceeding to the next step.

Before placing the sample in the scanning electron microscope (Basic Protocol 6), make sure that the paraffin has been removed by washing with toluene (Basic Protocol 5). It is necessary to observe the sample under an upright microscope several times and continue washing until you are sure.

The effectiveness of the protocols and procedures described here depend largely on following the details provided in each section and correctly implementing all steps.

Be systematic in labeling your samples, ensuring that the labels on the containers do not get washed off when changing solutions. Prepare labels in advance and work regularly to reduce the chance of errors.

We have not tested these protocols in other plant species; however, in principle, this set of protocols is likely applicable to the dry stems of other monocots, with adjustments in the storage time of the samples in solutions depending on the biology of each plant species. For protocol development, we avoided issues even for very dry and old samples. This set of protocols can easily be extended to thousands of case studies.

### Troubleshooting

Please refer to Table 3 for potential problems encountered during the preparation of cross‐sections and their corresponding solutions.

**Table 3:** Troubleshooting Guide for Obtaining Smooth Cross‐Sections

Problem	Possible cause	Solution
The paraffin block breaks during cutting	Basic Protocol 2 is not well implemented	Use a sharp blade and try to heat the area around the sample (to a degree that does not melt the paraffin), for example, use two reading lights around the microtome. If the problem is not resolved, repeat Basic Protocol 2.
Part of the sample is not cut	The entire surface of the sample is not in contact with the microtome blade	Re‐trim the sample, checking the angle of the specimen relative to the blade to ensure that it is perpendicular to the blade. Make a few preliminary cuts with the microtome before the main cuts.
The paraffin is cut, but not the sample	The blade is not sharp enough	Replace the disposable blade or clean its surface thoroughly with alcohol to remove any traces of paraffin from previous cuts. After placing it in the device and before cutting, make sure the blade is sufficiently warm.

### Understanding Results

Using the protocols presented here, dry and fragile rice plant stem tissues can be prepared optimally for SEM imaging. Tissues, such as stems, can be regenerated despite distorted cell topography and shape (Fig. 1B). High‐quality images can be obtained from years‐old specimens, opening up the possibility of using samples that were previously impossible to image due to their fragility and high risk of pulverization. Important aspects, such as the surface of the cell wall, and different types of cells and structures, including parenchymal cells, middle lamella, and vascular structures, can be studied in detail, without artificial or undesirable particles or distorted shapes.

As crucial as capturing high‐resolution, intact microscopic images for visual comparison of two different samples is, converting these qualitative observations into quantitative information is equally important in today's digital world. By providing the pipeline described in the image quantification protocol (Basic Protocol 7), it is possible not only to visually display the differences between two different samples in terms of the anatomical features of the cell wall but also to numerically compare them and provide quantitative documentation of the significance of these differences.

For comparison, different parts of the rice stem cell wall can be measured, such as the thickness of the stem cell wall; the distances between vascular bundles; the length of the parenchymal cell section; the thickness of the epidermal and sclerenchymal sections of the cell wall; the area of ​​the vascular section of the cell wall, including the area of ​​the xylem and phloem; and the area of ​​the midrib. In some cases, the number of cells constituting each section (such as parenchymal cells) can be counted on a specific scale (Fig. 8). Finally, the contribution of each of the sections in determining the thickness of the cell wall, which serves as a key indicator of the mechanical strength of the stem and reduces the likelihood of lodging, is notable.

SEM is reliant on the high‐magnification study of surfaces. If internal tissues need to be imaged, the samples should either be cut in the proper orientation or examined with other microscopy instruments, such as a transmission electron microscope (TEM) (Bello et al., 2017).

### Time Considerations

This is the first report on preparing dry plant samples for generating high‐quality images. Relative to standard SEM protocols, the procedure presented here is relatively easy, reproducible, and applicable to a wide array of tissues while optimally managing material reuse. There are specific steps that, if necessary, allow the process to be paused for an extended period without damaging the samples or compromising previous steps. In terms of cost, much of the equipment (e.g., glassware and micro‐holders) and consumables, such as paraffin, can be reused for multiple preparations. The reagents are inexpensive chemicals available from virtually all commercial suppliers.

The disadvantages of this method are that it is time consuming, requires a waste management procedure for proper disposal of chemicals such as aldehydes and ethanol, and lacks a key for data analysis (Bello et al., 2017).

Given that simple and readily available solutions (such as 70% ethanol) are often used to perform the entire preparation process, the main amount of time required to perform each protocol is not for making the solutions but rather for incubating the sample in those solutions. The duration is ∼30 days for Basic Protocol 1 and ∼8 days for Basic Protocol 2. The time required to prepare each paraffin block containing the sample, cut it, deparaffinize it, and prepare it for imaging (Basic Protocols 2 to 5) is 1 hr, 5 min, and 10 min, respectively, for steps 1, 2, and 3 and 4 combined. The time required to coat the sample with gold and place it in the scanning electron microscope for image capture (Basic Protocol 6) takes a total of 20 min. It should be noted that if you intend to use gelatin slides (Support Protocol), the preparation time required is 48 hr. Processing and quantification of each image (Basic Protocol 7) takes an average of 5 min.

### Author Contributions

**Mahta Mohamadiaza**: Formal analysis; investigation; writing—original draft. **Naser Farrokhi**: Conceptualization; supervision; writing—review and editing. **Pär K. Ingvarsson**: Resources; writing – review and editing. **Asadollah Ahmadikhah**: Conceptualization; supervision; writing—review and editing. **Mehdi Jahanfar**: Writing – review and editing.

### Conflict of Interest

The authors declare no conflict of interest.

## Data Availability

There are no data to be shared beyond what is presented in this article. All data generated or analyzed are included in this published article.
